# Outcomes of Non-surgical Management of Zygomaticomaxillary Complex Fractures

**DOI:** 10.1007/s12663-023-01863-1

**Published:** 2023-02-04

**Authors:** S. Arun, Sunil S. Nayak, A. Chithra, Sreea Roy

**Affiliations:** Department of Oral and Maxillofacial Surgery, Manipal College of Dental Sciences Manipal, Manipal Academy of Higher Education, Manipal, India

**Keywords:** Non-surgical mangement, ZMC fractures, Displaced fractures

## Abstract

**Introduction:**

With little evidence available in the literature, this study tries to clinically determine the efficiency and outcomes of non-surgical management of post-traumatic Zygomaticomaxillary complex (ZMC) fractures.

**Materials and Methods:**

One hundred and three patients with post-traumatic isolated ZMC fractures managed conservatively for various reasons were identified. The patients were classified based on the Zingg et al. criteria into Types A, B, and C. We evaluated the resolution of signs and symptoms of six standard parameters over 6 months—persistent pain, restriction in mouth opening, infraorbital nerve (ION) paresthesia, aesthetic deformity, infraorbital step deformity with associated tenderness on palpation, and ophthalmic status. The study variables were then statistically analyzed using Cochran’s Q test with an associated confidence interval of 95%.

**Results:**

A six-month follow-up revealed persisting residual deformities for all three groups. However, Type A and Type B showed significant improvement in pain reduction, mouth opening, and infraorbital nerve (ION) paresthesia. No significant improvement was noted in any of the groups for aesthetic deformity, infraorbital step deformity, and ophthalmic status. Type C, which had comminuted fracture patterns, exhibited significant defects in all the parameters. Significant inter-variable relationship between certain paired parameters was also observed.

**Conclusion:**

The Type A group is most suited for non-surgical management. Type B with a mono-bloc fracture is a crucial group that demands broader, long-term studies to extract a proper treatment protocol. Type C with severe fracture displacement validates surgical correction.

## Introduction

Zygoma is a robust bone forming the critical anterolateral surface of the middle third of the face. It is crucial in transmitting the occlusal stress toward the skull base. The convex prominence rationalizes its propensity for fractures. The optimal treatment modality for Zygomaticomaxillary complex (ZMC) fractures is a subject of active discussion. The decision-making of whether to treat ZMC fractures surgically or conservatively is critical [1]. Traditionally non-surgical management is indicated in minimally displaced/undisplaced and incomplete fractures with no functional or aesthetic compromise. These patients are advised to have a soft diet for 6 weeks and are regularly monitored [2]. Aesthetic defects such as flattening of cheekbone or dimpling, functional defects such as mouth opening limitations, and ophthalmic complications (enophthalmos, diplopia, restricted eye movements, and hypoglobus) are indications for open reduction of ZMC fractures [3, 4]. Functional defects are an absolute indication for open reduction.

However, open reduction and fixation may not be a suitable interventional method in patients with poor neurologic status associated with trauma. Polytrauma or late presentation can also delay or defer the treatment of complex ZMC fractures. These cases with existing contraindications to surgery or deferred management unfold a window with vast possibilities. In the era of evidence-based medicine, such patients add to a valuable conservative management group. The outcome-based follow-up of such nonsurgically managed patients can contribute to the literature with evidence-based results. We try to utilize this, and such patients managed conservatively for distinct reasons were identified and followed up. The study classifies patients based on the Zingg et al. criteria [5]. This classification is simple, direct, and widely used by clinicians. In this classification, the ZMC fractures are classified into three types based on severity (Type A, B, and C) depending on the extent of the fracture. This study aims to determine the outcomes of non-surgical management of ZMC fractures based on Zingg’s classification.

## Materials and Methods

This prospective review includes 103 patients aged 18–70 diagnosed with post-traumatic ZMC fractures from November 2019 to December 2021. The study was conducted after obtaining Institutional Ethics Committee approval (IEC: 919/2019) and individual informed consent. Once evaluated clinically, the patients underwent a computed tomography (CT) scan.

The inclusion criteria included: 1) Isolated, undisplaced/mildly displaced ZMC fractures. 2) Isolated displaced ZMC fractures associated with traumatic neurologic, abdominal/thoracic injuries (requiring hospitalization exceeding 6 weeks. The exclusion criteria included: 1) ZMC fractures associated with other facial fractures (Lefort, nasal and mandibular fractures). 2) ZMC fractures with associated pathologies such as cysts and tumors of the jaw bones.

**Fig. 1:**
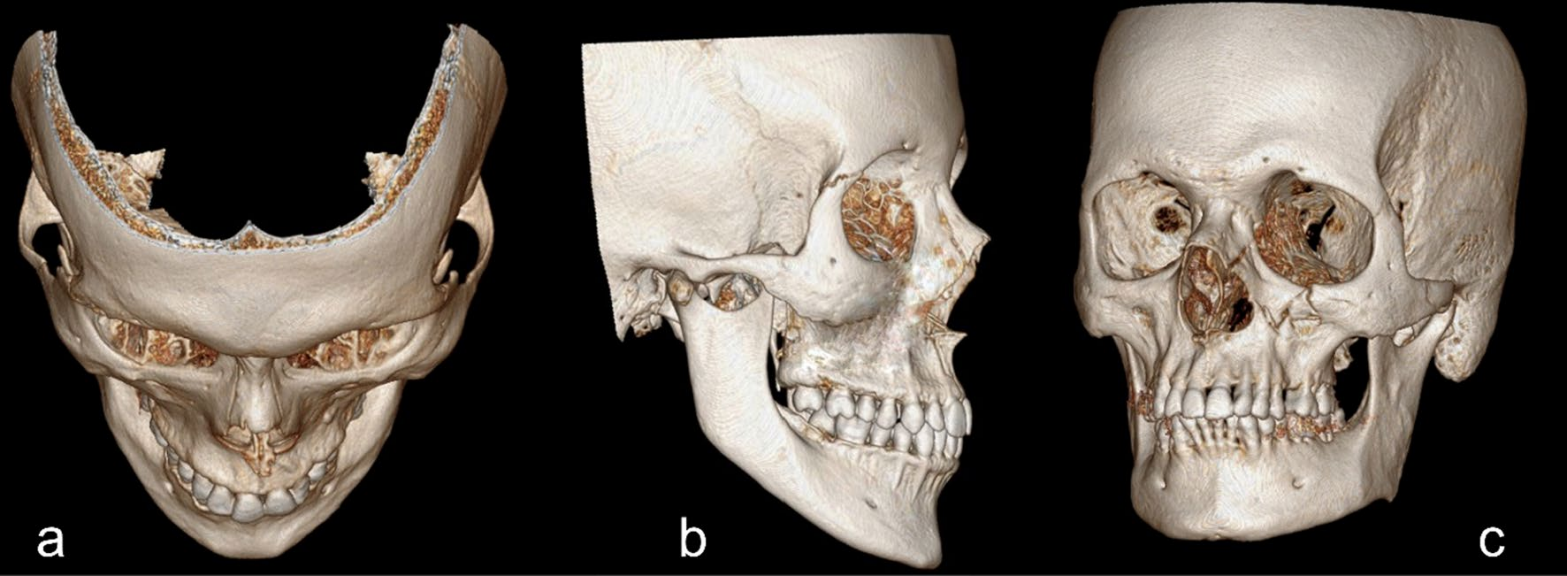
**a** Type A_1_—Isolated arch fracture, **b** Type A_2_—Lateral orbital wall fracture, **c** Type A_3_—Infra orbital rim fracture

**Fig. 2:**
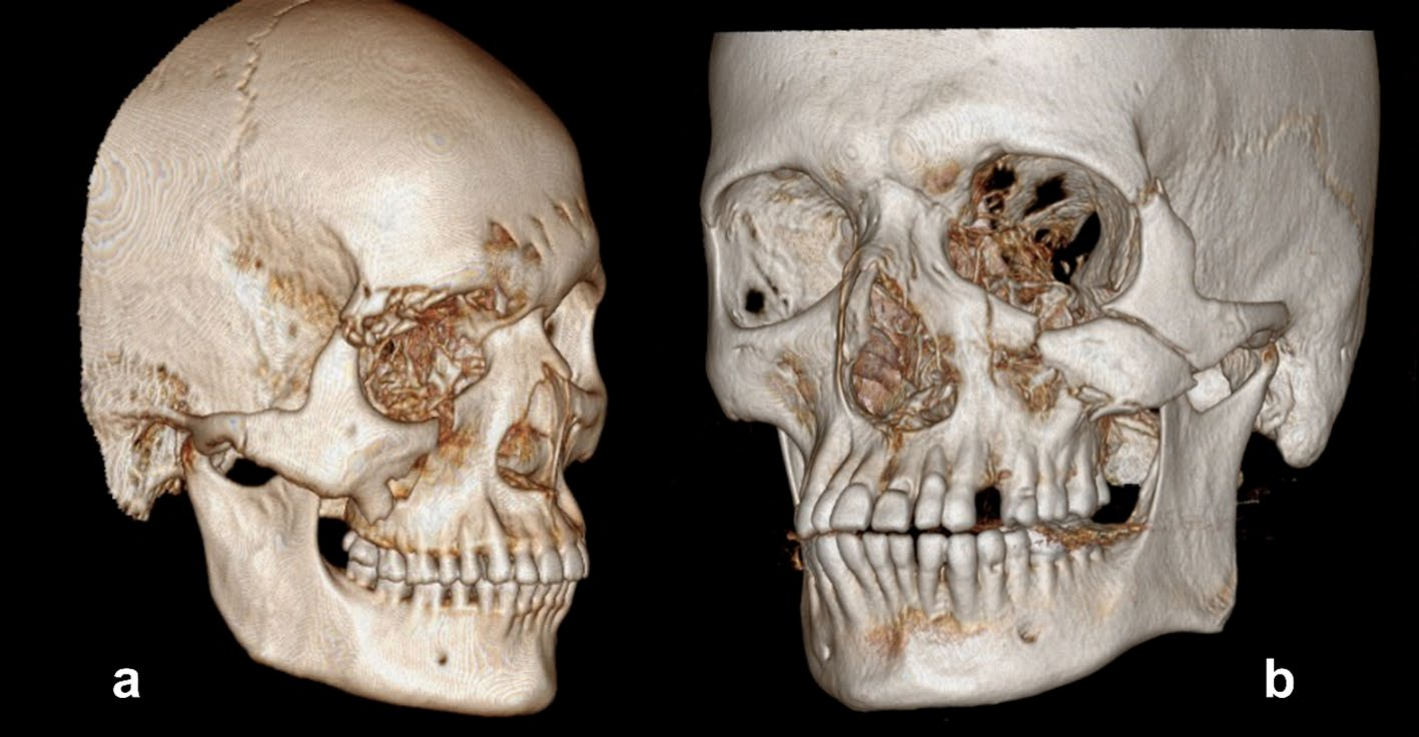
**a** Type B—Mono-bloc fracture, **b** Type C—Multi-fragment/comminuted fracture

The patients were divided based on the initial evaluation. They were classified using the Zingg et al. criteria into three groups: Type A with subtypes A_1_ (Isolated fracture of the arch), A_2_ (Fracture of the lateral orbital wall), and A_3_ (fracture of the infraorbital rim), Type B—complete mono-fragment type of fracture with separation at all sites of articulation and Type C—a multi-fragment type of fracture [5] (Figs. 1 and 2).

We carried out a single assessor evaluation system for the first visit (within 1 month) and at the 6-month follow-up post-trauma. The patients hospitalized with a low Glasgow Coma Scale (GCS) score during the initial presentation were followed up, and subjective data were collected once they were oriented and fit for evaluation. The subjects were evaluated based on the following six parameters: persistent pain, restriction in mouth opening, infraorbital nerve (ION) paresthesia, aesthetic deformity, infraorbital step deformity with associated tenderness on palpation, and ophthalmic status. At the end of 6 months, intergroup and intragroup comparisons were made using appropriate statistical tools. Using Cochran’s Q test and an associated confidence interval of 95%, the variables were analyzed with Jamovi (version 2.3). The non-surgical management included treating the patients symptomatically with antibiotics, analgesics, muscle relaxants, and active mouth opening physiotherapy.

## Results

Of the 110 patients identified for non-operative management, 103 were available for the follow-up period of 6 months. Of the 103 patients, 90 (87.3%) were males and 13 (12.6%) females. The average age of patients was 39.6 years (males 38.9, females 45.1). Sixty-two (60%) were under the influence of alcohol, and all were males. Road traffic accidents were the most common etiology (71 patients, 68.9%), followed by slip and fall (29 patients, 28.1%). Two patients (1.9%) were victims of interpersonal conflict, and 1 (0.97%) suffered from sports-related trauma. Seventy-seven patients (74.8%) sought medical attention within 24–96 h, while 26 (25.2%) presented 6–8 weeks post-trauma for management. Ninety-seven patients (94.1%) suffered from facial soft tissue injuries, of which 20 patients (20.6%) suffered from abrasions and 77 (79.4%) from deep lacerations.

The initial 1-month evaluation revealed pain to be the most common ailment (97.0%), while the least reported was ophthalmic-related complaints (11.7%) (Table 1). Forty-eight patients (46.6%) had Type A fractures, 44 patients (42.7%) had Type B, and only 11 patients (10.7%) suffered from Type C fractures (Table 2). Among Type A fractures, 33 patients (68.8%) had A_3_, 13 (27.0%) had A_1_, and A_2_ accounted for 2 patients (4.2%) (Table 2).

**Table 1:** Clinical assessment parameters and findings on first visit post-trauma

Assessment parameter	Number of patients (%)
Pain on chewing/biting	100 (97.0%)
Restriction in mouth opening	82 (79.6%)
Infraorbital nerve (ION) paresthesia	66 (64.0%)
Aesthetic deformity/malar flattening	54 (52.4%)
Tenderness on palpation of the infraorbital rim step deformity	48 (46.6%)
Ophthalmic condition (diplopia, pain in gazes)	12 (11.7%)

**Table 2:** Distribution of ZMC fractures based on Zingg et al. Classification

Type of fracture	Number of patients (%)
Type A	48 (46.6%)
A1	13 (27.0%)
A2	2 (4.2%)
A3	33 (68.8%)
Type B	44 (42.7%)
Type C	11 (10.7%)

On initial evaluation, 100 (97.0%) patients complained of pain in the ZMC region. Thirty-four (33.0%) patients continued to have pain in the final follow-up. Initially, 82 patients (79.6%) showed restriction in mouth opening, which reduced to 33 patients (32.0%) in the last follow-up (Table 3). Type A, with mild tissue injury, showed significant improvement in mouth opening after 6 months (75.8%), while Type C, with severe arch/muscle impingement, showed the least improvement (45.4%). The preliminary assessment revealed 66 patients (64.0%) suffering from ION paresthesia, which resolved to 27 patients (26.2%) in 6 months. For the above parameters, Type A and B groups showed statistically significant improvement (*P* < 0.001) compared to Type C after 6 months (Table 3).

**Table 3:** Follow-up evaluation—intra group

Assessment parameter (%)		Number of patients		Intra group improvement	
		First visit	6 months	Percentage	(*P* value < 0.05)
Pain on chewing/biting	A	46	9	80.4	< 0.001
	B	43	17	60.5	< 0.001
	C	11	8	27.3	1.000
Restriction in mouth opening < / = 30 mm	A	33	8	75.8	< 0.001
	B	38	19	50.0	< 0.001
	C	11	6	45.5	1.000
Infraorbital nerve (ION) paresthesia	A	22	5	77.3	< 0.001
	B	33	15	54.5	< 0.001
	C	11	7	36.4	1.000
Aesthetic deformity/Malar flattening	A	18	7	61.1	0.003
	B	27	17	37.0	0.510
	C	9	5	44.4	0.134
Tenderness on palpation of the Infraorbital rim step deformity	A	12	7	41.7	0.074
	B	28	18	35.7	0.385
	C	8	7	12.5	1.000
Ophthalmic deformity	A	1	1	00.0	1.000
	B	5	4	20.0	1.000
	C	6	5	16.7	1.000

Initial evaluation revealed 54 patients (52.4%) with aesthetic deformity, and the number decreased to 29 patients (28.2%) at the end of the study. Thirty-two patients (31.1%) had tenderness on palpation of the residual step deformity of the infraorbital rim on final evaluation compared to 48 (46.6%) during the initial review. During the final assessment, there was no resolution of the step deformity in any of the 48 cases. Twelve patients (11.7%) suffered from diplopia initially, and at the end of 6 months, 9 (8.7%) complained of persistent diplopia. On initial evaluation, one patient complained of pain in the upward and lateral eye gazes, which persisted after 6 months.

Inter-variable relationships between paired parameters (‘malar flattening and pain,’ ‘pain and restricted mouth opening,’ and ‘malar flattening and nerve deficit’) were statistically significant (Table 4).

**Table 4:** Follow-up evaluation—inter-variable relationship

Association between parameters	Inter-variable improvement—6 months (*P* value < 0.05)
Malar flattening and pain	< 0.001
Pain and restricted mouth opening	< 0.001
Malar flattening and infraorbital nerve paresthesia	< 0.001

## Discussion

Maxillofacial fractures are generally addressed for function, aesthetics, and quality of life. The trauma evaluation protocol varies among different centers, but the clinical parameters of concern are roughly the same.

We observed that the severity of trauma was more significant in patients under the influence of alcohol, thus increasing the risk of morbidity [6, 7]. This finding was more prevalent in the younger age groups (below 40 years). In the elderly, slip and falls were the primary causative factor, and injuries sustained could be attributed to age-related bone changes [8, 9]. Our study found a high incidence of facial soft tissue injuries associated with ZMC fractures. This was similar to the findings of Lim et al., who, in their study, found an association between facial fractures and concomitant injuries [10].

The decision to treat ZMC fractures surgically or conservatively is vital. Indications for surgical intervention include fracture displacement, malar depression, the presence of step deformity, or limited mouth opening [1, 11–13]. As per existing literature, mildly displaced fractures with minimal to no symptoms generally formed the criteria for conservative management of facial fractures [14]. But non-surgical treatment outcomes, especially of ZMC fractures, have rarely been discussed [14, 15]. In this study, we included patients with varying degrees of ZMC fracture based on the Zingg et al. classification. Due to varied reasons like associated neurological, abdominal, or thoracic injuries, some displaced fractures that traditionally warrant surgical intervention had to be managed conservatively in this study. Thus, for the first time, we have presented a comprehensive evaluation and enlisted the outcomes of non-surgical management of different grades of ZMC fractures.

Regarding the Zingg et al. classification, in our study, Type A fractures occurred more frequently than Type B but only marginally. Brucoli et al. (7) reported a higher incidence of Type B fractures, which may be attributed to their large sample size (1406). Among Type A fractures, the higher incidence of A3 and low incidence of A2 in our study similar to that of Brucoli et al. (7). The lower incidence of A2 fractures indicates that isolated fractures of the lateral orbital wall rarely occur. The low incidence of Type C fractures may be because patients in this group often seek surgical intervention to improve obvious functional and aesthetic deformities [16, 17].

Pain in the affected area reduces significantly over time [4]. Neuropathic pain can lead to chronic pain, which can affect the patient psychologically and influence the individual’s quality of life [18]. In our study, Type A had an 80.4% improvement after 6 months, followed by Type B (60.5%). We noticed that in non-surgical management, the lesser the fracture severity and displacement, the better the prognosis for pain reduction. The multi-fragmented Type C fracture group showed only a 27.3% improvement in pain reduction after 6 months (Table 3). These findings were similar to a study by Dubron et al., who reported a higher incidence of neuropathic pain symptoms in Type B and C fractures [18].

Restriction in mouth opening posed a significant concern for us. Thirty-three patients in the final evaluation, thirty (90.9%) did not report any restriction until clinical evaluation. This may be attributed to the dietary modifications made to cope with the same and the lack of awareness about the minimal requisites of a standard mouth opening. Coronoid fracture or impingement of the zygomatic arch is the usual cause of restricted mouth opening [19]. In our study, some fractures involving the zygomatic arch also showed restriction in mouth opening. The restriction may also be due to the damage of anatomical soft tissue attachments [1].

Altered nerve deficit is a well-documented phenomenon following fractures of the ZMC region [7]. Authors have attributed this to the involvement of the infraorbital canal/foramen within the ZMC fracture line, which acts as a “chink in one’s armor” [20, 21]. The vulnerability of this anatomic landmark leads to traction–compression or rupture of the nerve resulting in paresthesia [22, 23]. In minimally displaced fractures, quick resolution happens once edema or the hematoma settles [4, 24]. Ironically, surgically managed groups of patients too may suffer from nerve injury due to iatrogenic causes [25]. However, it is not discussed as it is beyond the scope of this study. As noted by Back et al., “there is no justification for surgical treatment of these fractures if altered nerve sensitivity is the only complication” [14]. In our study, while the infraorbital rim fracture (A3) is the most prevalent Type A fracture, its minimal displacement resonates with maximum improvement (77.3%) in 6 months. Significant correlations between Type B and increased nerve paresthesia were noted. 54.5% of patients reported resolution, while the rest continued to have paresthesia. These findings bear similarity to the study by Brucoli et al., who said that increased severity of the mono-bloc displaced ZMC fracture results in increased nerve paresthesia [7]. Type C, with possible nerve rupture, exhibited a minor resolution (36.4%) (Table 3).

Aesthetic deformity with or without malar flattening is a thoroughly documented finding [20] and is of aesthetic concern to patients. Some patients reported it affecting their personal or professional life. This could be due to the purely subjective nature of the deformity [26, 27]. Post-traumatic malar edema masks this deformity during the initial evaluation. Comparable improvement was noted in the Type A and B groups during the final review after 6 months but was not statistically significant (Table 3). The improvement was evident in only those cases of aesthetic deformity not associated with malar flattening.

One of the most frequent signs of a ZMC fracture is step deformity of the bony margin [28]. There can be associated tenderness on palpation of this region. No significant resolution of tenderness on palpation of the step deformity and the persistence of the step in all our reported cases suggests the possible need for surgical intervention (Table 3). Studies have similarly indicated that infraorbital rim deformity is one of the factors affecting prognosis and warrants surgical intervention for the same [1, 20].

In our study, diplopia was a noted complication, with one patient reporting painful gazes. Most subjects reporting diplopia were late presentation cases following trauma (more than 2 months). Popular literature documents diplopia as one of the common complications post ZMC fractures [7]. One patient had sought an ophthalmology consultation and is currently on prescription glasses. We found pain during lateral gazes in one patient, which persisted even after 6 months. There may be involvement of the III, IV, or VI cranial nerve, which warrants a complete ophthalmology evaluation [7]. Nevertheless, in Type B or C, it is essential to recognize the entrapment of the inferior rectus muscle into the orbital floor, which would warrant prompt surgical management [1]. The results of our study justify this fact, as no significant improvement of the ophthalmic condition was noted in any of the groups after 6 months (Table 3).

In our study, Type A benefitted the most from non-surgical management. We observed satisfactory improvement in parameters such as pain reduction, mouth opening, and infraorbital nerve paresthesia. Isolated fractures with minimal displacement could have aided adequate healing with time. Type A with nerve/muscle injury, infraorbital step, or diplopia exhibited slight improvement. Even mild fractures can lead to lasting deformity if not thoroughly diagnosed.

In Type B fractures, the mono-bloc zygoma is separated from its surrounding articulating bones. The deformities depend on the degree of displacement. It is known that the lesser the displacement, the better the non-surgical outcomes. However, few cases with appreciable fracture separation had satisfactory results with time, as noted with the improvement of pain, nerve paresthesia, and mouth opening. Self-healing capability is vital, but such observation demands long-term research for justification. Large sample size is required to identify suitable candidates for conservative management. This factor is a limitation of the present study.

This study reveals that Type C with comminution shows no significant improvement in any parameters. (*P* > 0.05). The severity of the fracture in this group demands surgical intervention to restore function and aesthetics.

Another notable observation was significant inter-variable relationships (Table 4). As one variable among the pair improves, the other factor improves and vice versa. These inter-variable relationships could give the surgeon insight and help decide to wait and watch or intervene promptly for the patient’s overall well-being.

## Conclusion

We can conclude that patients with undisplaced/mildly displaced fractures or single fractures (Type A) of ZMC are the best candidates for non-surgical management. Long-term research on mono-bloc Type B fractures corroborating various clinical parameters need to be carried out to understand better when to treat it nonsurgically. Irrespective of the Type, patients with malar flattening, infraorbital rim step deformity, and ophthalmic complications are likely to benefit from surgical intervention. Type C with a severe displacement of fracture fragments warrants surgical management.
